# Removal of Fast Flowing Nitrogen from Marshes Restored in Sandy Soils

**DOI:** 10.1371/journal.pone.0111456

**Published:** 2014-10-29

**Authors:** Eric L. Sparks, Just Cebrian, Sara M. Smith

**Affiliations:** 1 Dauphin Island Sea Lab, Dauphin Island, Alabama, United States of America; 2 Marine Sciences, University of South Alabama, Mobile, Alabama, United States of America; Beijing Forestry University, China

## Abstract

Groundwater flow rates and nitrate removal capacity from an introduced solution were examined for five marsh restoration designs and unvegetated plots shortly after planting and 1 year post-planting. The restoration site was a sandy beach with a wave-dampening fence 10 m offshore. Simulated groundwater flow into the marsh was introduced at a rate to mimic intense rainfall events. Restoration designs varied in initial planting density and corresponded to 25%, 50%, 75% and 100% of the plot area planted. In general, groundwater flow was slower with increasing planting density and decreased from year 0 to year 1 across all treatments. Nevertheless, removal of nitrate from the introduced solution was similar and low for all restoration designs (3–7%) and similar to the unvegetated plots. We suggest that the low NO_3_^−^ removal was due to sandy sediments allowing rapid flow of groundwater through the marsh rhizosphere, thereby decreasing the contact time of the NO_3_^−^ with the marsh biota. Our findings demonstrate that knowledge of the groundwater flow regime for restoration projects is essential when nutrient filtration is a target goal of the project.

## Introduction

Marsh restoration is a ubiquitous practice for mitigation of global marshland loss [1]. However, marsh restoration is expensive and labor intensive [2], [3]. Compounded with the costly nature of marsh restoration, there is often inconsistency and discrepant outcomes among different techniques and designs [3]. Some studies have been conducted to evaluate cost-effectiveness of vegetative growth for restored marshes [2]; however, evaluations of the ecosystem services provided by different marsh restoration designs is scant, but should be evaluated to inform managers interested in maximizing the effectiveness of restoration projects [4]–[6].

Marshes provide important ecosystem services [7]–[10], and it has been suggested that nutrient filtration is the most economically valuable ecosystem service [4]. Processes such as denitrification and plant uptake can remove a large portion of nutrient inputs into marshes as groundwater percolates through the marsh rhizosphere [11], [12].

Most nutrient filtration studies for marshes are conducted in mature natural marshes that are subjected to low to moderate flows of groundwater [12]–[14]. These studies have demonstrated that the presence of marsh plants increases nutrient removal through direct plant uptake as well as facilitating bacterial processes responsible for outgassing nitrogen (e.g., denitrification and anammox; [15]). However, marshes are subjected to varying groundwater flow rates from upland sources [16]–[18] and are dependent on factors such as rainfall intensity and soil permeability. In general, when areas are subjected to intense flow events (e.g., heavy rain), it is likely a smaller portion of the nutrients carried in these events can be removed than when the site is subjected to lower flow rates [19]. Along the northern Gulf of Mexico (nGOM) coast, there are frequent and intense rain events [20], thereby subjecting these marshes to a mixture of fast flow events, during and immediately after these rain events, and lower flow between events [16]. Assessment of nutrient removal by restored marshes under different scenarios of groundwater flow is important to improve the effectiveness of marsh restoration efforts targeting nutrient filtration as a primary goal.

In this study, we use black needlerush (*Juncus roemerinaus*) as our restored marsh plant. Black needlerush marshes are dominant on the nGOM coast [21] and have suffered significant loss over past decades primarily attributed to coastal development [22]. Due to the losses of marshes along the nGOM coast and prevalence of black needlerush, this marsh plant is the target for many restoration projects [2], [23]–[25].

In this study, we compare groundwater flow rate and nitrate (NO_3_^−^) removal from an introduced groundwater solution in five black needlerush marsh restoration designs, varying in initial plant density, with unvegetated controls immediately after planting and one year after planting. Utilization of these marsh planting designs allows for comparisons of NO_3_^−^ removal from fast flowing groundwater across designs that vary in the effort required to plant (i.e., time and cost). The groundwater plume introduced into the marsh mimics a pulse of groundwater derived from an intense rainfall event percolating through porous sediments. Expectations were groundwater flow rates, through the marsh rhizosphere, would decrease and NO_3_^−^ removal would increase with increasing planting density over time. Results from this study can inform managers interested in maximizing restoration efficiency with the goal of reducing nutrient pollution into water bodies.

## Materials and Methods

### 1. Site Construction

**Figure 1:**
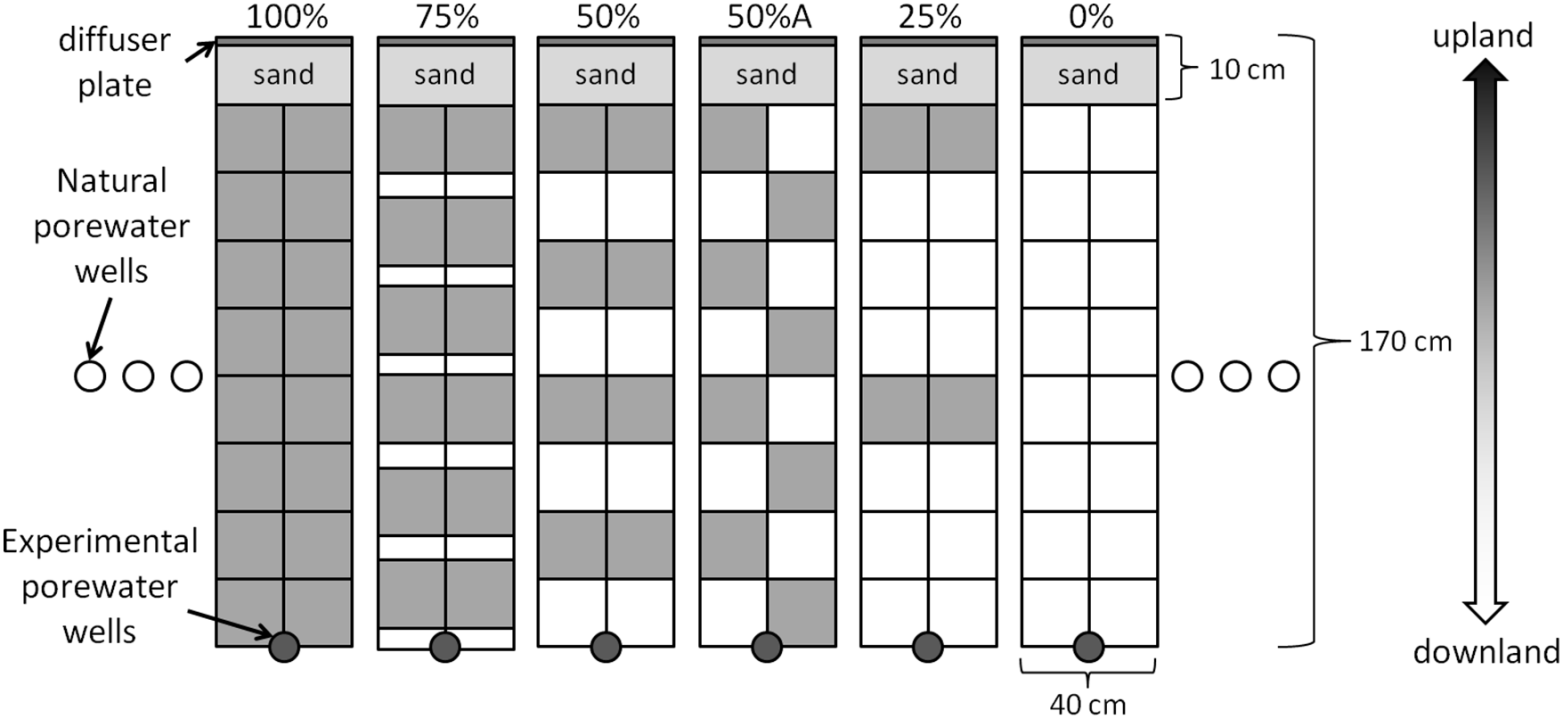
Schematic of 1 block of 6 marsh restoration designs (0%, 25%, 50%, 50%A, 75% and 100%). Shaded squares represent planted sods. There were a total of 3 blocks with each block consisted of a randomized arrangement of all 6 restoration designs. The groundwater solution was introduced at the diffuser plate and flowed down the plots toward the porewater collection well.

On June 11, 2010, we planted a black needlerush marsh on the outskirts of Camp Beckwith (30°23′16″ N, 87°50′31″ W) located on the eastern coast of Weeks Bay in Fairhope, Alabama, USA. The staff of this privately owned camp gave us permission to conduct this work on their property and they should be contacted for future permissions. The planting site was situated on a stretch of sandy beach with natural marsh nearby. This sandy beach was subjected to high wave energy from boat wakes. To reduce wave action at this site, a fence was constructed, prior to marsh restoration, ten meters offshore of the restoration site to reduce shoreline wave energy and erosion. The fence consisted of a wooden frame filled up with dead tree branches and trunks along with other natural debris. Black needlerush sods (approximately 20 cm long, 20 cm wide and 20 cm deep) were harvested from an adjacent marsh and planted at the restoration site. Individual sods had a black needlerush shoot density typical of nGOM salt marshes, with ranges from 1400–1800 shoots m^−2^ [2]. For experimental setup, we used a randomized block design with 3 blocks consisting of 6 plots each, yielding a total of 18 experimental plots (Fig. 1). Blocks were separated by 2 m and each plot had dimensions of 40 cm wide and 170 cm long (Fig. 1). Plots represented different restoration designs in terms of initial plant density (25%, 50%, 50%A, 75% and 100%) plus non-vegetated controls (0%) rendering 6 designs with 3 replicated plots each. Each plot contained 16 sod-sized units and the number of sods planted in the plot corresponded to the planting density treatment (e.g., 4 sods for the 25% planting density; Fig. 1). Plots planted in the 50%A design were arranged in an alternating “checkerboard” pattern (Fig. 1). To contain the introduced groundwater solution, each plot was enclosed on the top (i.e., upland) and the two lateral sides with vertical placement of rigid plastic sheeting. Thirty cm of the sheet height was buried below the sediment surface with 10 cm of sheet height above the sediment surface. Porewater collection wells, screened from 5 cm to 30 cm below the sediment surface, were placed at the bottom of each plot (experimental porewater wells) and 3 (natural porewater wells) on each lateral side of every block (Fig. 1). A diffuser plate was buried in the sand 10 cm from the upland planting edge to help disperse the introduced solution.

### 2. Experimental methodology and sampling

On June 21, 2010 and June 30, 2011 fluorescein tracing tests were conducted by releasing 15 L of a 20 mg L^−1^ fluorescein solution into each plot. Fluorescein was used because it is not actively removed through biological processes at high rates; therefore, the only factor that can change the fluorescein concentration is dilution [26]. These attributes of fluorescein allow it to be used to assess travel time and dilution rates of groundwater plumes through the plots [26]. The fluorescein solution was released during receding tides when the tide line was at the upland edge of the plots. It took approximately 15 min for the 15 L of solution to disperse through the diffuser plate. This quick pulse was intended to mimic groundwater inputs from an intense rain event over porous sediments and was equivalent to a 1.5 cm rainfall event drained off of a 1 hectare area through 100 linear meters of fringing marsh over a 12 hour period. Porewater samples were taken from each well in 15 min intervals after tracer release for 90 minutes and stored on ice in a cooler for transport back to Dauphin Island Sea Lab for analysis. All fluorescein samples were analyzed on a Turner Designs-700 fluorometer.

To determine how effective the marsh designs were at removing nutrient pollution from a fast flowing groundwater solution, four rounds of NO_3_^−^ solution release and subsequent sampling were performed after the initial planting (June 27, July 8, 9, and 13, 2010) and 1 year post-planting (July 14, 21, 29 and August 2, 2011). The releases were done during receding tides when the tide line was at the upland edge of the plots (i.e., the same timing as the fluorescein tracing tests). For each round we released 15 L of a 200 µM NO_3_^−^ solution into each plot. Water samples were taken from each input container and porewater well at the bottom of the plots. The sampling timeline for porewater samples was determined by the fluorescein experiments (i.e., sample at time fluorescein peak for each plot). To determine background nutrient levels, we also took samples from the natural porewater wells located outside of each block on every sampling day. Porewater samples were filtered in the field and transported to the lab on ice for analysis. NO_3_^−^ concentrations were analyzed using cadmium reduction azo dye assays [27].

### 3. Calculations and statistical analyses

#### 3.1. Flow rates

Flow rates were analyzed by recording the time when peak fluorescein concentration was observed in each plot. These peak times were analyzed using an ANOVA (treatment × year × block). If block and the interaction between treatment and year were found to be insignificant factors, data were pooled across block and reanalyzed with an ANOVA (treatment × year). For all statistical analyses, tests were conducted using Sigma Stat 3.5 and significance was considered at p<0.05 [28].

#### 3.2. NO_3_^−^ removal

**Figure 2:**
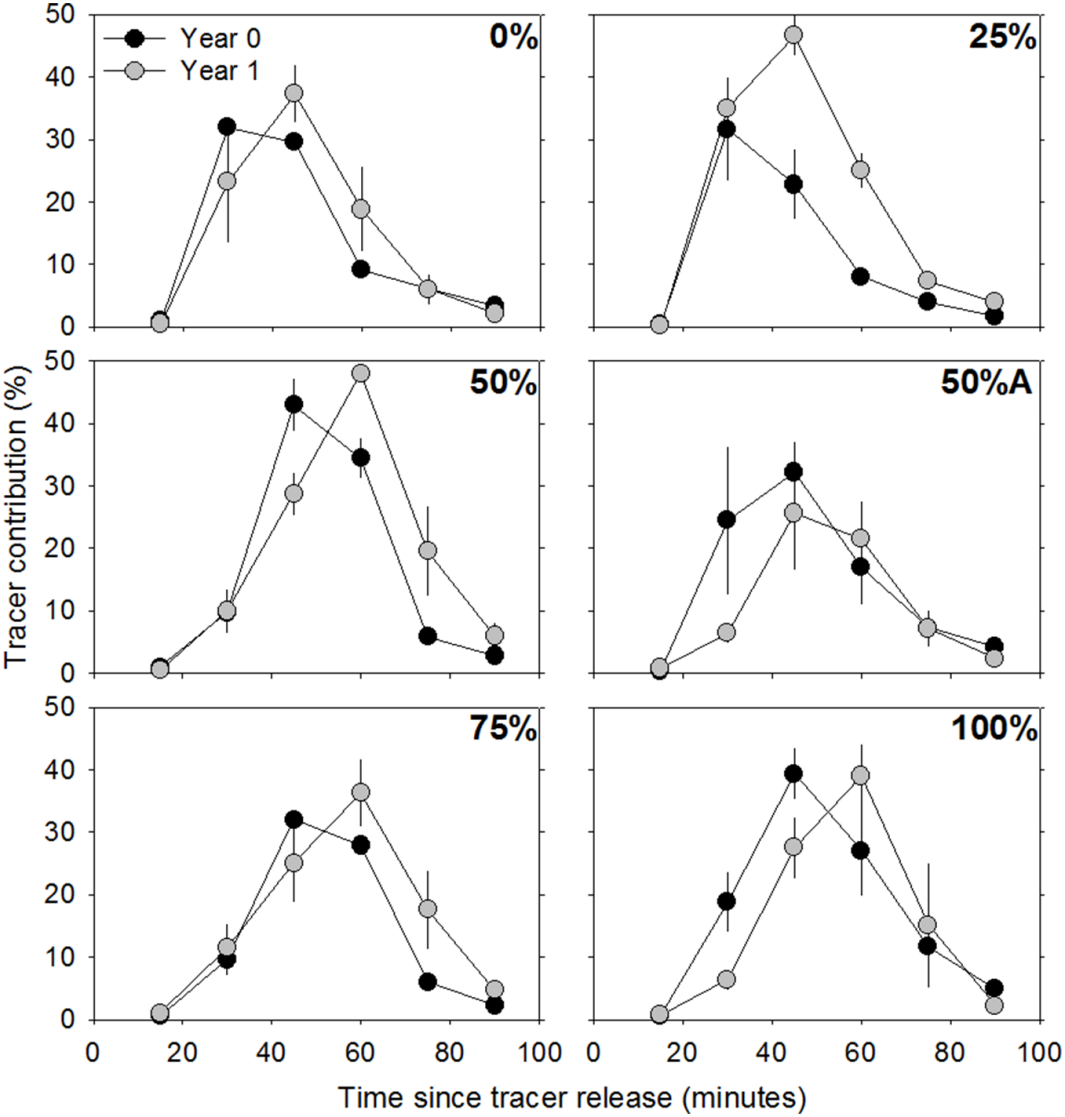
Fluorescein tracer porewater contribution (%) at the downland well through time. Black circles represent year 0 and grey circles represent year 1 samples. Percentages in the top right portion of each plot represents the planting density (0%, 25%, 50%, 50%A, 75% and 100%). Error bars indicate ±1 SE.

As the introduced solution travels through the plots, it will be subjected to dilution through mixing with natural porewater (i.e., not subjected to the introduced solution) with lower background concentrations of NO_3_^−^. To determine the [NO_3_^−^] in the portion of the porewater derived from the introduced solution, a dilution correction must be applied that accounts for the [NO_3_^−^] found in natural porewater. Applying a dilution correction allows for the removal of NO_3_^−^ from the introduced solution to be calculated as the introduced solution (*Input*) travels to the downland edge of the marsh. To calculate removal of NO_3_^−^ from the introduced solution, we subtracted the dilution corrected [NO_3_^−^] at the downland porewater well from the NO_3_^−^ concentration in the input using the following equation: *Removal  =  Input - (Downland well – Natural × Dilution) ÷ (1-Dilution)*. The previous equation will be referred to as equation 1 throughout the manuscript. The term *Downland well* in equation 1 is the [NO_3_^−^] measured at restored marsh porewater wells subjected to the simulated pollution plume. The *Natural* term is the [NO_3_^−^] in the porewater wells outside of the restored marsh that was not subjected to the simulated pollution plume (Fig. 1). The *Dilution* term in equation 1 is the proportion of sample derived from natural porewater and was calculated as the proportional decrease in [fluorescein] from the input to the porewater collection well (i.e., peak % tracer contribution in Fig. 2). NO_3_^−^ removal was converted to percent removal by dividing it by [NO_3_^−^] of the input. Similar to the flow rate study, percent NO_3_^−^ removal was first analyzed using an ANOVA (treatment × year × block). If block and the interaction between treatment and year were found to be insignificant factors, the data was pooled across block and reanalyzed with an ANOVA (treatment × year).

## Results

For flow rate and NO_3_^−^ removal, block was never a significant factor (flow rate - p = 0.40; NO_3_^−^ removal - p = 0.14) and there were no significant interactions between treatment and year (flow rate - p = 0.34; NO_3_^−^ removal - p = 0.99). Therefore, data were pooled across blocks and analyzed with an ANOVA for the effects of treatment and time (Table 1). Only the results of the ANOVA on the data pooled across blocks will be further discussed.

**Table 1:** Results of ANOVA for flow rates and NO_3_^−^ removal.

Test	Effect	Degrees of Freedom	F value	P value
Flow rates	Treatment	5	21.545	<0.001
	Year	1	76.818	<0.001
	Treatment × Year	5	1.200	0.339
NO_3_^−^ removal	Treatment	5	1.195	0.317
	Year	1	0.197	0.658
	Treatment × Year	5	0.020	0.999

Most of the fluorescein solution traversed the plots in less than one hour across all treatments (Fig. 2). In general, the flow of the introduced solution decreased with increasing planting density (Table 1), as indicated by the later peaks in the dilution curves (Fig. 2). Furthermore, it took longer for the solution to cross the plots one year after planting than two weeks after planting (Table 1; Fig. 2). Longer retention time of the introduced solution within the plots at one year after planting than two weeks after planting implies that flow rates of the introduced solution, through the plots, decreased over time.

**Figure 3:**
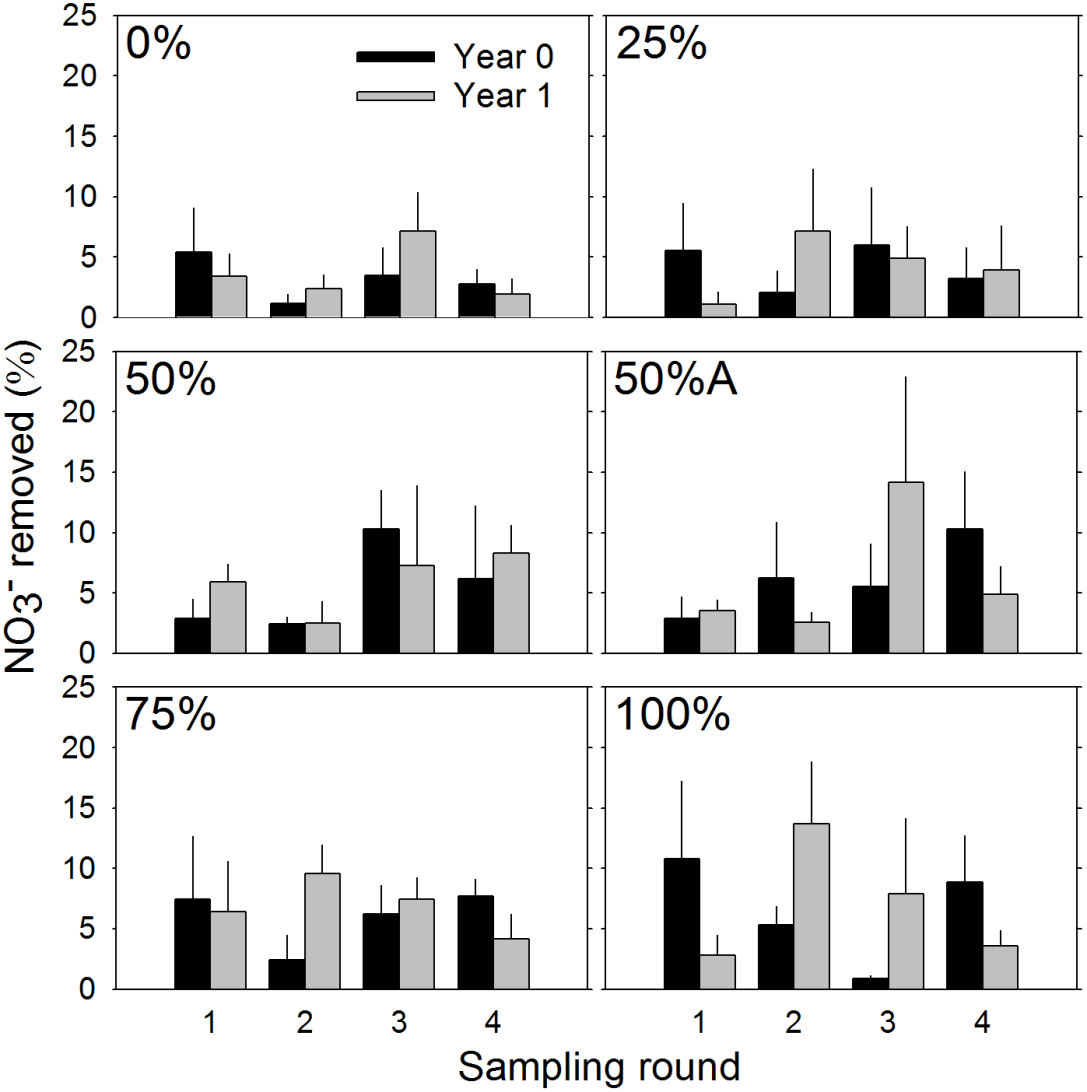
Percentage of NO_3_^−^ removed from introduced solution directly after planting and 1 year post-planting for each sampling round. Black bars represent year 0 and grey bars represent year 1 samples. Percentages in the top left portion of each plot represent initial planting densities. Error bars indicate ±1 SE.

Concentrations of NO_3_^−^ in porewater collections wells outside the plots ranged from 0.5 µM to 1.5 µM (i.e., natural or ambient porewater) and were low when compared to concentrations within the plots (i.e., subjected to the introduced solution). As expected, the input had [NO_3_^−^] of 200 µM±10 µM, whereas wells at the downland edge of the plots had lower [NO_3_^−^] ranging from 44 µM to 126 µM. When combining the observed changes in [NO_3_^−^] with a dilution factor (equation 1), our calculations showed only a small percentage of NO_3_^−^ was removed from the introduced solution (3–7%; Table 2). These small percentages of NO_3_^−^ removal were similar across all treatments (Table 1) and sampling years (Table 1; Fig. 3). While NO_3_^−^ processing increased slightly over time and with plant cover (Table 2) these differences were not statistically significant (Table 1).

**Table 2:** Mean percent NO_3_^−^ removal across 6 restoration designs directly after planting and 1 year after planting (±1 SE).

	Treatment					
Year	0%	25%	50%	50% A	75%	100%
0	3.22 (±0.87)	4.29 (±0.94)	5.44 (±1.81)	6.23 (±1.54)	5.95 (±1.22)	6.47 (±2.18)
1	3.73 (±1.19)	4.28 (±1.25)	5.99 (±1.27)	6.27 (±2.68)	6.89 (±1.12)	6.97 (±2.49)

## Discussion

In this study, we found that the introduced groundwater traveled slowest through the most vegetated planting designs and slower one year after planting than immediately after planting. These results offer evidence that the presence of marsh plants increases the time required for groundwater to flow through the restoration area, likely through the presence of the marsh rhizosphere and accumulation of finer grained sediments [29]. As the planted plots mature and density increases, they will likely continue to decrease groundwater flow rates through binding sediments, expansion of the marsh rhizosphere [30] and reduction of wave energy that aids in the accumulation of finer grained sediments [31].

Increases in plant density and the accumulation of finer grained sediment are conducive to increased nutrient removal in marshes [15]. We did find some suggestive evidence that increases in vegetated area increased NO_3_^−^ removal (Table 2), albeit not statistically significant (Table 1). Our range in planting density was large (0%–100%) and we measured NO_3_^−^ removal in all of these planting densities twice over one year. If planting density was a primary driver of NO_3_^−^ removal from this fast flowing solution, we would have captured it with this sampling design. Given no effect of planting density and overall findings that only a small portion of the input NO_3_^−^ was removed across all of the planting designs through time (3–7%), it appears that the groundwater was traveling too quickly through the marsh rhizosphere for plants to have an impact on NO_3_^−^ removal. In studies where groundwater traveled slower through the marsh rhizosphere, marsh plants had time to uptake and facilitate bacterial processes that fueled large removals of nitrogen [17]. Comparing these studies to our study suggests that flow rate can influence how effective marshes are at removing NO_3_^−^ from groundwater. With these results, it is likely that nutrient processing in our restored marsh will remain similar to the unvegetated plots for several years to come [5]. An additional factor contributing to the negligible increases in NO_3_^−^ removal over time is the typical slow growth of black needlerush [21]. This slow growth pattern was evident by visual observations of marginal increases in vegetated area (<5%) for the vegetated designs and no colonization of the 0% planting design at one year after planting. A timeframe when these marshes will decrease groundwater flow enough to allow for large proportions of input nutrients to be removed is unknown; however, previous studies have indicated restoring other ecosystem functions in restored marshes to natural levels takes many years [2], [32], [33].

A probable explanation to the high groundwater flow rates and low nutrient filtration is that the sediment at the restoration site was sandy, which is also the case for many restored marshes (e.g., Jamaica Bay marsh islands in New York, USA, Grand Bay National Estuarine Research Reserve boat launch marsh in Mississippi, USA, and the Labranche wetlands in Louisiana, USA). Groundwater flows more quickly through sand than sediments with higher mud and silt content typical of mature marshes [34]. Most other studies of nutrient filtration in salt marshes have been conducted mature natural marshes with fine sediments (mainly mud and silt). Finer sediments have smaller interstitial spaces and slow down groundwater flow in relation to the flow in sandy sediments [12]–[14], [35]. Slower flow rates increase contact time between nutrients and reactive areas of the sediment and rhizosphere, thereby increasing opportunities for nutrient processing [19]. In addition, sandy sediments are primarily composed of inorganic material (e.g. quartz) and typically contain little organic matter, which limits the biological processing of incoming nutrients [15].

While we did not find evidence for a strong role of these marsh planting designs as filters of runoff nutrient pollution, they provide other important services such as habitat and shoreline stabilization. We did not quantify these services, but we would expect these services to be small in magnitude for these young marshes and increase as the marshes age [6]. Similarly, we expect that these restored marshes will increase vegetated area and become effective nutrient filters through time.

## Conclusions

In conclusion, our results suggest that groundwater flow rates and sediment type should be considered when planning marsh restoration efforts with the specific goal of runoff pollution removal. Despite finding slower groundwater flow rates with increasing vegetation, our introduced groundwater did still flow quickly through the restored plots (30–60 minutes). This fast flow is most likely attributable to the coarse texture of the sediment. We calculated that on average only 3–7% of the NO_3_^−^ entering the restored marshes was processed by the marshes within one year since planting, and it appears that NO_3_^−^ processing by these restored marshes will remain similar to the unvegetated plots for many years to come. However, the slower groundwater flow in the more vegetated plots suggests they will likely become effective nutrient filters prior to the less vegetated plots. Understanding the nature, control, and extent of nutrient processing by restored marshes requires additional research, such as more restoration designs across many different marsh environments, in order to help managers decide on best restoration practices given budget constraints.

## Supporting Information

Data Set S1 Data for flow rate and dilution calculations.
(XLSX)

Data Set S2 Data for NO_3_^−^ removal calculations.
(XLSX)
